# A Rare Case of Idiopathic Chylopericardium Causing Cardiac Tamponade

**DOI:** 10.7759/cureus.81229

**Published:** 2025-03-26

**Authors:** Klodian Krakulli, Alessia Mehmeti, Arber Aliu, Manjola Liko, Henri Kolani, Ervin Bejko, Altin Veshti

**Affiliations:** 1 Cardiac Surgery, University Hospital Center "Mother Teresa", Tirana, ALB; 2 Cardiology, University Hospital Center "Mother Teresa", Tirana, ALB; 3 General Surgery, University Hospital Center "Mother Teresa", Tirana, ALB

**Keywords:** chylopericardium, diagnosis, etiology, management, medium-chain triglyceride diets, ocreotide, surgical treatment

## Abstract

Chylopericardium (CP) is a rare clinical entity characterized by the accumulation of chylous fluid within the pericardial space. It can be classified as primary (idiopathic) or secondary, with the latter associated with trauma, cardiothoracic surgery, malignancies, or infections. We present a case of a 67-year-old male patient with idiopathic CP who presented with signs of cardiac tamponade. The patient was initially managed conservatively with pericardiocentesis, dietary modifications, and octreotide but required surgical intervention due to persistent chyle drainage. This case highlights the diagnostic and therapeutic challenges associated with idiopathic CP and emphasizes the importance of early recognition and appropriate management.

## Introduction

Chylopericardium (CP) is an uncommon cause of pericardial effusion and is often underdiagnosed due to its rarity. First described by Hasebroek in 1888 [1], primary CP remains an enigmatic condition with no clearly identifiable cause. Secondary CP is more common and occurs as a result of lymphatic disruption due to trauma, surgery, malignancies, or infection [2-4]. Dib et al. proposed a scoring system to aid in diagnosing CP [5]. The score is based on the presence of the following criteria: (i) a milky yellowish fluid appearance, (ii) a triglyceride (TG) level exceeding 500 mg/dL, (iii) a total cholesterol (TC) to TG ratio of less than 1, and (iv) a negative bacterial culture with a lymphocytic predominance in the fluid cell count. Each criterion is assigned 1 point, and a total score of 2 is required for diagnosis, with both specificity and sensitivity reaching 100%. Treatment of CP consists of dietary modification, fluid removal, and surgery. Surgery is usually considered when the patient does not respond to conservative therapy [6].

A comprehensive literature review was conducted using databases such as PubMed, Scopus, and Web of Science. The search terms included “chylopericardium,” “etiology,” “diagnosis,” “management,” and “surgical treatment.” Articles from the past three decades were prioritized, with a focus on case reports, systematic reviews, and clinical guidelines.

For surgical approaches, we analyzed case series describing thoracic duct ligation, pericardial drainage, and dietary modifications as primary treatment strategies. Non-surgical management strategies, including the role of medium-chain triglyceride (MCT) diets, octreotide therapy, and percutaneous drainage, were also reviewed.

## Case presentation

A 67-year-old man with a history of smoking and hypertension presented with progressive dyspnea and shortness of breath for four months. He had been diagnosed with pericardial effusion three months prior at a local hospital and was treated with colchicine and non-steroidal anti-inflammatory drugs for over a month. Despite treatment, his symptoms worsened, and he was admitted to our emergency department with signs of cardiac tamponade.

Emergent pericardiocentesis in the intensive care unit (ICU) yielded 700 mL of milky white pericardial fluid. A pericardial catheter was placed for continuous drainage. Fluid analysis confirmed CP with a TG level >1500 mg/dL (reference range: 0-50 mg/dl), a predominance of lymphocytes, and a TG-to-TC ratio >1. Aerobic and anaerobic cultures were negative.

The patient was started on conservative management, including (NPO/nil per os) dietary status with the exception of medium-chain TGs, total parenteral nutrition (TPN), and subcutaneous octreotide (100 mcg every eight hours). The pericardial fluid gradually became clearer, and TG levels dropped below 100 mg/dL. However, persistent drainage exceeding 300 mL per day for over two weeks and a consistently high TG-to-TC ratio prompted surgical intervention.

The patient underwent thoracic duct ligation via right postero-lateral thoracotomy and partial pericardiectomy. Postoperatively, pericardial drainage was monitored, and oral lipid intake was reintroduced on the second day. No recurrence of chylous effusion was observed. The patient was discharged on postoperative day six and remained asymptomatic on follow-up.

**Figure 1:**
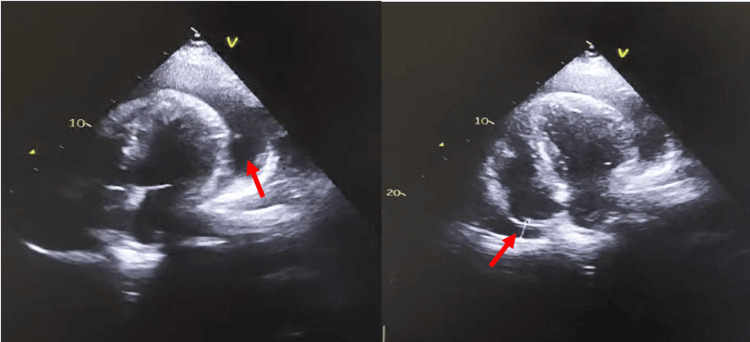
Preoperative transthoracic echocardiography showing circumferential pericardial effusion

**Figure 2:**
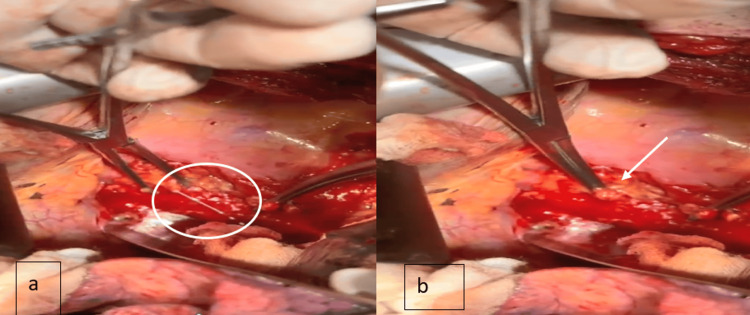
Operative images. a. The chyle leak from the thoracic duct. b. Ligation of the thoracic duct

**Figure 3:**
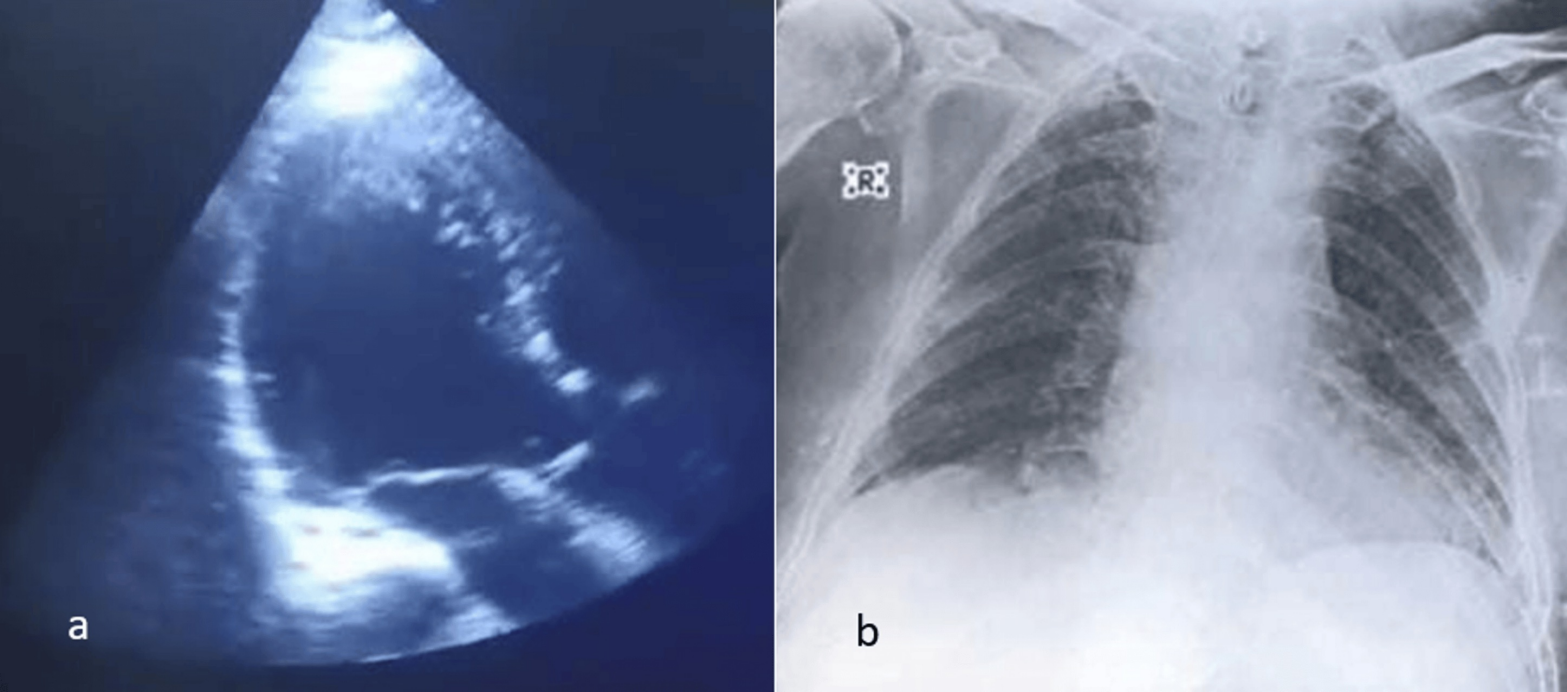
Postoperative images. a. Transthoracic echocardiography showing no pericardial effusion. b. Postoperative X-ray showing a normal heart size

Figure 1 shows the preoperative transthoracic echocardiography, with evident circumferential pericardial effusion; the intraoperative process of the thoracic duct ligation is shown in Figure 2, and the postoperative imaging shows no pericardial effusion (Figure 3).

## Discussion

CP is a rare condition that requires a high index of suspicion for diagnosis. It can be categorized as primary (idiopathic) or secondary. Idiopathic CP accounts for approximately 56% of cases and is believed to result from congenital abnormalities of the lymphatic system, impaired lymphatic valve function, or abnormal communications between lymphatic and pericardial vessels. Secondary CP, on the other hand, is usually due to trauma, cardiothoracic surgery, malignancy, infection, or thrombosis of the jugular vein [5,7].

The clinical presentation of CP is highly variable. Many patients remain asymptomatic and are diagnosed incidentally on imaging, while others present with nonspecific symptoms such as dyspnea, cough, chest pain, or syncope. Patients are commonly noted to have enlargement of the cardiac silhouette on routine chest radiography. This is followed by an echocardiogram, utilized to confirm the presence of a pericardial effusion and to assess for impairment of venous filling during diastole. Cardiac tamponade occurs in approximately 17-30% of cases and necessitates immediate intervention [8]. In our patient, worsening dyspnea and signs of tamponade prompted emergent pericardiocentesis, leading to the diagnosis.

Definitive diagnosis requires pericardiocentesis and fluid analysis. Chylous fluid is typically milky white with a TG level >500 mg/dL, a predominance of lymphocytes, and a TC-to-TG ratio <1. Confirmatory testing includes Sudan III staining for fat globules and negative bacterial cultures. Further evaluation with computed tomography and lymphangiography is essential to identify potential secondary causes. Lymphangiography remains the gold standard for mapping the thoracic duct and detecting lymphatic leaks [8,9].

Management of CP depends on the severity of symptoms and underlying etiology. Conservative treatment is attempted first, especially in idiopathic cases, and includes pericardiocentesis, dietary modifications, and pharmacological agents like octreotide. Octreotide reduces chyle production by inhibiting gastrointestinal secretion, splanchnic blood flow, and thoracic duct output [10]. It is important to note that various studies have concluded that conservative treatment in CP fails in approximately 50% of the cases [7,11]. In our patient, conservative therapy led to an initial improvement, with a reduction in TG levels and a change in fluid appearance. However, persistent drainage and an unchanged TG-to-TC ratio indicated the need for surgical intervention.

Surgical treatment is advised when pericardial drainage exceeds 1,500 mL per day, remains above 500 mL per day after five days, or leads to malnutrition [12]. Thoracic duct ligation via VATS is the preferred surgical approach, as it carries a low risk of morbidity and has no long-term nutritional consequences. Pericardiectomy is performed to ensure complete drainage and to prevent secondary constrictive pericarditis. The preferred and most widely recommended surgical approach by most surgeons is ligation of the thoracic duct above the diaphragm [13]. In our case, the patient underwent thoracic duct ligation, partial pericardiectomy, and pericardial window formation via postero-lateral thoracotomy, leading to complete resolution of symptoms.

Long-term prognosis after surgical intervention is favorable, with a recurrence rate of less than 5% [11]. The review published by Dib et al. also cited that surgical management was curative for all patients with CP [5]. However, close monitoring is necessary, as persistent lymphatic leaks can lead to complications such as malnutrition, immunodeficiency, and recurrent effusions. Early recognition and appropriate management significantly impact patient outcomes.

In the literature, there are approximately 120 cases of CP described, but one of the major reviews is the one performed by Yu et al. [7], where 104 cases from 92 studies were gathered and analyzed. This major study found that a total of 62 cases initially underwent conservative management, which included dietary control through TPN, a low-fat diet, and an MCT diet. Among these, 36 cases did not respond and required surgery, while 26 cases improved with conservative treatment. Overall, surgery was performed in 71 cases (71.2%). A pericardial window was created in 50 cases (48.08%), thoracic duct ligation was carried out in 46 cases (44.23%), and embolization was performed in four cases (3.85%). Only one case underwent reconstruction of the thoracic duct, which is rarely performed.

Follow-up data were available for 64 cases, with a median follow-up duration of 12 months (ranging from one month to nine years). All patients had favorable outcomes, with only one case developing constrictive pericarditis. There were no fatalities during the follow-up period. In conclusion to this study, it was agreed that surgical management is the most successful treatment and is associated with a favorable prognosis [7].

Our case, as well as several ones published in international literature, highlights the diagnostic and therapeutic challenges of idiopathic CP. Given its rarity, it is often misdiagnosed or treated as a non-specific pericardial effusion. A structured approach involving pericardiocentesis, fluid analysis, imaging, and, when necessary, surgical intervention is crucial for optimal management.

## Conclusions

CP is a rare but significant cause of pericardial effusion and cardiac tamponade. Early diagnosis requires a high index of suspicion and confirmation with fluid analysis. Initial conservative management can be effective but fails in more than half of the cases. Surgical intervention is often necessary for persistent or recurrent cases. Thoracic duct ligation and pericardial window formation remain the gold-standard treatment options. Although more case reports are needed for a clearer understanding, the available data suggest that CP is a treatable condition with excellent surgical outcomes and favorable long-term follow-up results.
